# The estimation of the Burr-XII parameters with middle-censored data

**DOI:** 10.1186/s40064-015-0856-3

**Published:** 2015-02-28

**Authors:** Ali H Abuzaid

**Affiliations:** Department of Mathematics, Al-Azhar University-Gaza, Gaza, Palestine

**Keywords:** Coverage percentage, Gamma distribution, Lindley’s approximation

## Abstract

Middle-censoring is considered as a modern general scheme of censoring. In this paper, we study the analysis of middle-censored data with Burr-XII distribution which is considered one of the most popular and flexible distributions for modeling stochastic events and lifetime for many products.

The parameters are estimated by the maximum likelihood method and the Bayes estimation under gamma prior and by applying the Lindley’s approximation.

A simulation study is carried out to compare the performances of the two estimates. Both estimators behave almost similarly and verified the consistency property. A real medical data set is considered for illustration.

## Introduction

Burr (1942) constructed a system of distributions that contains twelve types. The Burr-XII distribution denoted by Burr-XII (*a*, *b*) is one of the most popular distributions due to its appropriateness for modelling stochastic events (Zimmer et al. 1998) and its flexibility for representing the lifetime for many products where it has a non-monotone hazard function (Soliman 2002). Furthermore, Burr–XII curve can cover the curve shape characteristics for different distributions including normal, exponential, Weibull, logistic, log normal and extreme value type I distribution (see Wang et al. 1996).

The probability density function and the cumulative distribution function of the Burr-XII distributed random variable with shape parameter *a* and scale parameter *b* are given by:

$$ \begin{array}{cccc}\hfill f(t)= ab{t}^{b-1}{\left(1+{t}^b\right)}^{-a-1},\hfill & \hfill t>0,a>0,b>0,\hfill & \hfill \mathrm{and}\hfill & \hfill F(t)=1-{\left(1+{t}^b\right)}^{-a},\hfill \end{array} $$

respectively. Wang et al. (1996) discussed the maximum likelihood estimation of complete and censored data. On the other hand, several authors considered the Bayesian estimation of other types of Burr distributions under complete and with different censoring schemes (see Abd-Elfattah and Alharbey 2012; Feroze and Aslam 2012).

In this paper, a general censoring scheme, known as Middle-censoring as presented in Middle-censoring, is considered to obtain the estimation of the Burr-XII parameters with middle-censored data.

This paper is organized as follows: Middle-censoring reviews the definition and literature in the middle-censoring. Maximum likelihood estimation presents the maximum likelihood estimation, the approximated asymptotic variance-covariance matrix and the confidence interval. In Bayes estimation, we provide the Bayesian formulation and explain the Lindley’s approximation of the posterior expectation. The numerical results of the simulation studies on the performances of the two estimators are presented in Simulation results, and an illustrative example on a medical data set is given in Data analysis.

## Middle-censoring

Jammalamadaka and Mangalam (2003) proposed a general censoring mechanism called the middle-censoring scheme in non-parametric set up and is differentiated from other censoring schemes. Middle-censoring occurs if a data point is not observable when it falls inside a random interval. Suppose *T*_1_, …, *T*_*n*_ are the lifetimes of *n* identical items. For the *i*th item, there is a random censoring interval (*L*_*i*_, *R*_*i*_) with some unknown bivariate distribution. Exact value of *T*_*i*_ is observable only if *T*_*i*_ ∉ [*L*_*i*_, *R*_*i*_], otherwise the actual value is not observable, but we observe the interval (*L*_*i*_, *R*_*i*_).

Iyer et al. (2008) claimed that left-censoring, right-censoring and double-censoring schemes can be obtained as special cases of this middle-censoring scheme by suitably choosing censoring intervals, which can be infinite. Furthermore, they illustrated that middle-censoring is not a complementary to the idea of double-censoring where a random middle part is missing.

Middle-censoring may arise in several situations as presented by Jammalamadaka and Mangalam (2003). In any lifetime study, we have an interval of censorship if the subject is temporarily withdrawn from the study. It can be a patient under observation may be absent from study for a short period during which time the event of interest may occur. Equipment failure that could occur during a period where the observation is not possible or is not being made.

Iyer et al. (2008) applied the idea of middle-censoring to the analysis of data from exponential lifetime distributions, and more recently, Bennett (2011) explored middle-censoring for further parametric models like the Weibull and gamma families and extended it to parametric models with covariates.

In this paper, we analyze the Burr-XII lifetime data when they are middle-censored. Assume that *T*_1_, …, *T*_*n*_ are *i.i.d* Burr-XII (*a*, *b*) random variables. Let *Z*_*i*_ = *R*_*i*_ − *L*_*i*_, *i* = 1, …, *n* to be another random variable defines the length of the censoring interval with exponential distribution with mean *γ*^-1^, where the left-censoring point for each individual *L*_*i*_ is assumed to be also an exponential random variable with mean *λ*^-1^. Moreover, the ${T}_i^{\prime }s,\kern0.6em {L}_i^{\prime }s$ and ${Z}_i^{\prime }s$ are all independent of each other and the observed data, ${X}_i^{\prime }s$ are given by $$ {X}_i=\left\{\begin{array}{c}\hfill \begin{array}{cc}\hfill {T}_i\hfill & \hfill \begin{array}{cc}\hfill if\hfill & \hfill {T}_i\notin \left({L}_i,{R}_i\right),\hfill \end{array}\hfill \end{array}\hfill \\ {}\hfill \begin{array}{cc}\hfill \left({L}_i,{R}_i\right)\hfill & \hfill \begin{array}{ccc}\hfill \hfill & \hfill otherwise.\hfill & \hfill \hfill \end{array}\hfill \end{array}\hfill \end{array}\right. $$

## Maximum likelihood estimation

Suppose that *n* randomly selected units from Burr-XII (*a*, *b*) population, where *a* and *b* are both unknown, are put on test under middle-censoring scheme. To write up the likelihood function, assume that there are *n*_1_ > 0 uncensored observations and *n*_2_ > 0 censored observations. Then, without loss of generality, by re-ordering the observed data into the uncensored and censored observations. Therefore, we have the following data $\left\{{T}_1,\dots, \kern0.5em {T}_{n_1},\kern0.5em \left({L}_{n_1+1},\kern0.5em {R}_{n_1+1}\right),\dots, \kern0.5em \left({L}_{n_1+{n}_2},{R}_{n_1+{n}_2}\right)\right\}\kern0.1em ,$ where *n*_1_ + *n*_2_ = *n*. Thus, the likelihood function of the observed data is given by:

3.1 $$ L\left(a,b\left|t\right.\right)=c{(ab)}^{n_1}{\displaystyle \prod_{i=1}^{n_1}{t}_i^{b-1}{\left(1+{t}_i^b\right)}^{-\left(a+1\right)}{\displaystyle \prod_{i={n}_1+1}^{n_1+{n}_1}\left[{\left(1+{r}_i^b\right)}^{-a}-{\left(1+{l}_i^b\right)}^{-a}\right]}}, $$

where *c* is a normalizing constant depending on *λ* and *γ*. The estimation of *λ* and *γ* is not of interest, thus it is left as a constant. The log-likelihood function is given by

$$ \begin{array}{l}l\left(a,b\left|t\right.\right)= \log c+{n}_1 \log a+{n}_1 \log b+{\displaystyle \sum_{i=1}^{n_1} \log {t}_i^{b-1}}-\left(a+1\right){\displaystyle \sum_{i=1}^{n_1} \log \left(1+{t}_i^b\right)}\\ {}\kern4em +{\displaystyle \sum_{i={n}_1+1}^{n_1+{n}_1} \log \left[{\left(1+{r}_i^b\right)}^{-a}-{\left(1+{l}_i^b\right)}^{-a}\right]},\end{array} $$

The maximum likelihood estimation (MLE) of *a* and *b* denoted by *â*_*M*_ and ${\widehat{b}}_M$ can be derived by solving the following equations, respectively

3.2 $$ \begin{array}{l}\frac{\partial l\left(a,b\left|t\right.\right)}{\partial a}=\frac{n_1}{a}-{\displaystyle \sum_{i=1}^{n_1} \log \left(1+{t}_i^b\right)}\\ {}\kern4em -{\displaystyle \sum_{i={n}_1+1}^{n_1+{n}_1}\frac{{\left(1+{r}_i^b\right)}^{-a} \log \left(1+{r}_i^b\right)-{\left(1+{l}_i^b\right)}^{-a} \log \left(1+{l}_i^b\right)}{\left[{\left(1+{r}_i^b\right)}^{-a}-{\left(1+{l}_i^b\right)}^{-a}\right]}},\end{array} $$

and

3.3 $$ \begin{array}{l}\frac{\partial l\left(a,b\left|t\right.\right)}{\partial b}=\frac{n_1}{b}+{\displaystyle \sum_{i=1}^{n_1} \log {t}_i}-\left(a+1\right){\displaystyle \sum_{i=1}^{n_1}\frac{t_i^b \log {t}_i}{1+{t}_i^b}}\\ {}\kern4em -{\displaystyle \sum_{i={n}_1+1}^{n_1+{n}_1}\frac{a{\left(1+{r}_i^b\right)}^{-\left(a+1\right)}{r}_i^b \log {r}_i-a{\left(1+{l}_i^b\right)}^{-\left(a+1\right)}{l}_i^b \log {l}_i}{\left[{\left(1+{r}_i^b\right)}^{-a}-{\left(1+{l}_i^b\right)}^{-a}\right]}},\end{array} $$

It is obvious that the MLE of *a* and *b* cannot be solved explicitly. Therefore, the solutions could be obtained by using Newton–Raphson method, or numerically by using the solve systems of nonlinear equations “*nleqslv*” package in R.

The asymptotic variance-covariance of the MLE for parameters *a* and *b* are given by the elements of the inverse of the Fisher information matrix

$$ {I}_{ij}=-E\left(\frac{\partial^2l\left(a,b\left|t\right.\right)}{\partial a\partial b}\right),\kern1em i,j=1,2. $$

The approximate asymptotic variance-covariance matrix for the MLE will be considered because the exact mathematical expression for the above expectation is very difficult to obtain. Therefore, the approximate asymptotic variance-covariance matrix is given by

3.4 $$ \widehat{\varSigma}={\left[\begin{array}{cc}\hfill -\frac{\partial^2l\left(a,b\left|t\right.\right)}{\partial {a}^2}\hfill & \hfill -\frac{\partial^2l\left(a,b\left|t\right.\right)}{\partial a\partial b}\hfill \\ {}\hfill -\frac{\partial^2l\left(a,b\left|t\right.\right)}{\partial b\partial a}\hfill & \hfill -\frac{\partial^2l\left(a,b\left|t\right.\right)}{\partial {b}^2}\hfill \end{array}\right]}_{a={\widehat{a}}_M,b={\widehat{b}}_M}^{-1}=\left[\begin{array}{cc}\hfill {\widehat{\sigma}}_a^2\hfill & \hfill {\widehat{\sigma}}_{ab}\hfill \\ {}\hfill {\widehat{\sigma}}_{ab}\hfill & \hfill {\sigma}_b^2\hfill \end{array}\right] $$

with

$$ \begin{array}{l}\frac{\partial^2l\left(a,b\left|t\right.\right)}{\partial {a}^2}=\frac{n_1}{a^2}-{\displaystyle \sum_{i={n}_1+1}^{n_1+{n}_1}\frac{x_{r_i}^{-a}{x}_{l_i}{\left[{s}_{r_i}^{-a}-{s}_{l_i}^{-a}\right]}^2}{A^2}},\\ {}\frac{\partial^2l\left(a,b\left|t\right.\right)}{\partial a\partial b}=\frac{\partial^2l\left(a,b\left|t\right.\right)}{\partial b\partial a}=-{\displaystyle \sum_{i=1}^{n_1}\frac{t^b \log {t}_i}{\left(1+{t}^b\right)}}+\frac{r_i^b{q}_{r_i}{x}_{r_i}^{-a-1}\left[-1+a{s}_{r_i}\right]-{l}_i^b{q}_{l_i}{x}_{l_i}^{-a-1}\left[-1+a{s}_{l_i}\right]}{A}\\ {}\kern4.5em -\frac{\left(-a{r}_i^b{q}_{r_i}{x}_{r_i}^{-a-1}+a{l}_i^b{q}_{l_i}{x}_{l_i}^{-a-1}\right)\left({x}_{r_i}^{-a}{s}_{r_i}+{x}_{l_i}^{-a}{s}_{l_i}\right)}{A^2},\end{array} $$

and

$$ \frac{\partial^2l\left(a,b\left|t\right.\right)}{\partial {b}^2}=-\frac{n_1}{b^2}-\left(a+1\right){\displaystyle \sum_{i=1}^{n_1}\frac{t_i^b{\left( \log {t}_i\right)}^2}{{\left(1+{t}_i^b\right)}^2}}+{\displaystyle \sum_{i={n}_1+1}^{n_1+{n}_1}\frac{Y}{A^2}} $$

where following re-parameterization is used for the simplicity of expression presentations

$$ {x}_{l_i}=1+{l}_i^b,\kern1em {x}_{r_i}=1+{r}_i^b,\kern1em {q}_{l_i}= \log {l}_i,\kern1em {q}_{r_i}= \log {r}_i,\kern1em {s}_{l_i}= \log \left(1+{l}_i^b\right),\kern1em {s_r}_i= \log \left(1+{r}_i^b\right),A={x}_{r_i}^{-a}-{x}_{l_i}^{-a} $$

and

$$ \begin{array}{l}Y=a\left(a+1\right)\left\{{q}_{r_i}{x}_{r_i}^{-2a-2}\left[{q}_{r_i}\left(1+2{r}_i^b\right)-{x}_{r_i}^{-a}{x}_{l_i}^{-a}\right]+{q}_{l_i}{x}_{l_i}^{-2a-2}\left[{q}_{l_i}\left(1+2{l}_i^b\right)-{x}_{l_i}^a{x}_{r_i}^{-a}\right]\right\}\\ {}\kern1em +{\left[a{r}_i^b{q}_{r_i}{x}_{r_i}^{-a-1}-a{l}_i^b{q}_{l_i}{x}_{l_i}^{-a-1}\right]}^2.\end{array} $$

Since the MLE is asymptotically normal, thus the approximate confidence intervals for the parameters *a* and *b* can be computed as follows ${\widehat{a}}_M\pm {z}_{\frac{\alpha }{2}}\sqrt{{\widehat{\sigma}}_a^2}$ and ${\widehat{b}}_M\pm {z}_{\frac{\alpha }{2}}\sqrt{{\widehat{\sigma}}_b^2}$ where ${z}_{\frac{\alpha }{2}}$ is the value of the standard normal curve and *α* is the level of significance.

## Bayes estimation

This section considers the Bayesian formulation of the problem of estimating the scale and shape parameters of lifetime data from Burr-XII (*a*, *b*) with middle-censoring. Since *a* and *b* are both unknown, we will assume that the parameter *b* has an exponential distribution with mean 1/ *β* and the prior density of *b* is given by *π*_1_(*b*) = *βe*^− *βb*^ for *b*, *β* > 0, while the parameter *a* given the parameter *b* has a gamma prior distribution with shape parameter *θ* and scale parameter *b*. The conditional density function of *a* given *b* for *b*, *θ* > 0 is given by:

4.1 $$ {\pi}_2\left(a\left|b\right.\right)=\frac{b^{\theta }}{\varGamma \left(\theta \right)}{a}^{\theta -1}{e}^{- ba}, $$

Then the bivariate prior density function for a natural choice of the prior distributions of *a* and *b*, is assumed to be in the following form:

4.2 $$ \pi \left(a,b\right)={\pi}_1(b){\pi}_2\left(a\left|b\right.\right). $$

No prior distribution on the censoring parameters is assumed. Combining (4.1) and (4.2) the joint posterior density of *a* and *b* is given by:

4.3 $$ \begin{array}{l}\pi \left(a,b\left| data\right.\right)\\ {}\kern3em =\frac{\beta {a}^{n_1+\theta -1}{b}^{n_1+\theta }{e}^{-b\left(a+\beta \right)}}{\varGamma \left(\alpha \right)}{\displaystyle \prod_{i=1}^{n_1}{t}_i^{b-1}{\left(1+{t}_i^b\right)}^{-\left(a+1\right)}{\displaystyle \prod_{i={n}_1+1}^{n_1+{n}_1}\left[{\left(1+{r}_i^b\right)}^{-a}-{\left(1+{l}_i^b\right)}^{-a}\right].}}\end{array} $$

Under the Squared Error Loss (SEL) function, $L\left(\varphi, \widehat{\varphi}\right)={\left(\varphi -\widehat{\varphi}\right)}^2$, The Bayes estimator of a function *U* = *U* (*a*, *b*), *Û*_*s*_ is the posterior expectation given as

4.4 $$ {\widehat{U}}_s=E\left(U\left(a,b\right)\left| data\right.\right)=\frac{{\displaystyle \underset{0}{\overset{\infty }{\int }}{\displaystyle \underset{0}{\overset{\infty }{\int }}U\left(a,b\right)\pi \left(a,b\left| data\right.\right)\kern0.5em da\;db}}}{{\displaystyle \underset{0}{\overset{\infty }{\int }}{\displaystyle \underset{0}{\overset{\infty }{\int }}\pi \left(b,a\left| data\right.\right)\;da\;db}}} $$

There is no closed form of the ratio of the two integrals in (4.4). Lindley (1980) proposed asymptotic approximation to evaluate the ratio of two integrals. It can be expressed to parameters in following form:

4.5 $$ \begin{array}{l}{\widehat{U}}_s=E\Big[U\left({\varphi}_1,{\varphi}_2\right)\left| data\Big]\right.=U\left({\theta}_1,{\theta}_2\right)+\frac{1}{2}{\displaystyle \sum_{i=1}^2{\displaystyle \sum_{j=1}^2{u}_{ij}{\varepsilon}_{ij}}}+\frac{1}{2}{L}_{30}\left({u}_1{\varepsilon}_{11}+{u}_2{\varepsilon}_{12}\right){\varepsilon}_{11}\\ {}\kern1.5em +\frac{1}{2}{L}_{21}\left(3{u}_1{\varepsilon}_{11}{\varepsilon}_{12}+{u}_2\left({\varepsilon}_{11}{\varepsilon}_{22}+2{\varepsilon}_{12}^2\right)\right)+\frac{1}{2}{L}_{12}\left(3{u}_2{\varepsilon}_{22}{\varepsilon}_{21}+{u}_1\left({\varepsilon}_{11}{\varepsilon}_{22}+2{\varepsilon}_{21}^2\right)\right)\\ {}\kern1.5em +\frac{1}{2}{L}_{03}\left({\varepsilon}_{22}{u}_2+{\varepsilon}_{21}{u}_1\right){\varepsilon}_{22}.\end{array} $$

where ${u}_i=\frac{\partial U}{\partial {\varphi}_i},i=1,2,\kern1em {u}_{ij}=\frac{\partial^2U}{\partial {\varphi}_i\partial {\varphi}_j},i=1,2,\kern0.5em {L}_{ps}=\frac{\partial^{p+s}L}{\partial {\varphi}_1^p\partial {\varphi}_2^s},\kern0.5em \mathrm{f}\mathrm{o}\mathrm{r}\kern0.5em p,s=0,..,3$ and *p* + *s* = 3. Furthermore, *ε*_*ij*_ are the elements of the inverse of the matrix having elements {−*L*_*ij*_}, where *L* is the log likelihood of the joint prior and ${L}_{ij}=\frac{\partial^2L}{\partial {\varphi}_i\partial {\varphi}_j},\kern0.5em i,j=1,2.$

Now by applying the Lindley’s approximation into our case where (*φ*_1_, *φ*_2_) = (*a*, *b*) and

4.6 $$ \begin{array}{l}L= \log \pi \left(a,b\left| data\right.\right)\\ {}\kern1em = \log \beta +\left({n}_1+\theta -1\right) \log a+\left({n}_1+\theta \right) \log b-b\left(a+\beta \right)- \log \varGamma \left(\theta \right)\\ {}\kern2em +\left(b-1\right){\displaystyle \sum_{i=1}^{n_1} \log {t}_i}-\left(a+1\right){\displaystyle \sum_{i=1}^{n_1} \log \left(1\_{t}_i^b\right)}+{\displaystyle \sum_{i={n}_1+1}^{n_1+{n}_2} \log \left[{\left(1+{r}_i^b\right)}^{-a}-{\left(1+{l}_i^b\right)}^{-a}\right]}\end{array} $$

where all the terms are evaluated at the MLE *â*_*M*_ and ${\widehat{b}}_M$. The values of *L*_*ps*_, for *p*, *s* = 0, 1, 2, 3 can be obtained as following

$$ {u}_1=\frac{n_1+\theta -1}{a}-b-{\displaystyle \sum_{i=1}^{n_1} \log \left(1+{t}_i^b\right)}-{\displaystyle \sum_{i={n}_1+1}^{n_1+{n}_2}\frac{x_{r_i}^{-a}{s}_{r_i}-{x}_{l_i}^{-a}{s}_{l_i}}{A}}, $$

$$ {u}_2=\frac{n_1+\theta }{b}-\left(a+\beta \right)+{\displaystyle \sum_{i=1}^{n_1} \log \left({t}_i\right)-\left(a+1\right)}{\displaystyle \sum_{i=1}^{n_1}\frac{t_i^b \log {t}_i}{\left(1+{t}_i^b\right)}}-a{\displaystyle \sum_{i={n}_1+1}^{n_1+{n}_2}\frac{x_{r_i}^{-a-1}{q}_{r_i}{r}_i^b-{x}_{l_i}^{-a-1}{q}_{l_i}{l}_i^b}{A}}, $$

$$ {L}_{30}=\frac{2\left({n}_1+\theta -1\right)}{a^3}-{\displaystyle \sum_{i={n}_1+1}^{n_1+{n}_2}\frac{x_{l_i}^{-a}{x}_{r_i}^{-a}\left[{x}_{r_i}^{-a}{s}_{l_i}+3{x}_{r_i}^{-a}{s}_{r_i}+{x}_{l_i}^{-a}{s}_{l_i}-{x}_{l_i}^{-a}{s}_{r_i}\right]{B}^2}{A^3},} $$

$$ {L}_{21}=-{\displaystyle \sum_{i={n}_1+1}^{n_1+{n}_2}\frac{\left({x}_{l_i}^{-a-1}{x}_{r_i}^{-a-1}\right)\left\{{B}^2\left(-a\;{l}_i^b{q}_{l_i}{x}_{r_i}\left[1+{x}_{l_i}^{-a}\right]-a\;{r}_i^b{q}_{r_i}{x}_{l_i}\left[1+{x}_{r_i}^{-a}\right]\right)+2B\left(\;{r}_i^b{q}_{r_i}{x}_{l_i}-{l}_i^b{q}_{l_i}{x}_{r_i}\right)\right\}}{A^2}}, $$

$$ \begin{array}{l}{L}_{03}=\frac{2\left({n}_1+\theta \right)}{b^3}-\left(a+1\right){\displaystyle \sum_{i=1}^{n_1}\frac{t_i^b{\left( \log {t}_i\right)}^3}{{\left(1+{t}_i^b\right)}^2}}+2a{\displaystyle \sum_{i={n}_1+1}^{n_1+{n}_2}\frac{\left[{q}_{r_i}{r}_i^b{x}_r^{-a-1}-{q}_{l_i}{l}_i^b{x}_l^{-a-1}\right]\left\{{J}_{r_i}-{J}_{l_i}\right\}}{A^2}}+{\displaystyle \sum_{i={n}_1+1}^{n_1+{n}_2}\frac{F^2}{A^3}}\\ {}\kern1em -{\displaystyle \sum_{i={n}_1+1}^{n_1+{n}_2}\frac{a{q}_{r_i}^3\left[\left(a+1\right)\left(a+2\right){r}_i^{3b}{x}_{r_i}^{-a-3}-2\left(a+1\right)\;{r}_i^{2b}{x}_{r_i}^{-a-2}+{r}_i^b{x}_{r_i}^{-a-1}-\left(a+1\right){r}_i^{2b}{x}_{r_i}^{-a-2}\right]}{A}}\\ {}\kern1em +{\displaystyle \sum_{i={n}_1+1}^{n_1+{n}_2}\frac{a{q}_{l_i}^3\left[\left(a+1\right)\left(a+2\right){l}_i^{3b}{x}_{l_i}^{-a-3}-2\left(a+1\right)\;{l}_i^{2b}{x}_{l_i}^{-a-2}+{l}_i^b{x}_{l_i}^{-a-1}-\left(a+1\right){l}_i^{2b}{x}_{l_i}^{-a-2}\right]}{A}}\\ {}\kern1em +{\displaystyle \sum_{i={n}_1+1}^{n_1+{n}_2}\frac{a^2{q}_{r_i}{r}_i^b{x}_r^{-a-1}{J}_{r_i}+{a}^2{q}_{l_i}{l}_i^b{x}_l^{-a-1}{J}_{l_i}-\left(a{q}_{r_i}{q}_{l_i}{r}_i^b{l}_i^b{x}_{r_i}^{-a-1}{x}_{l_i}^{-a-1}\right)\left[\left(a+1\right)\;\left({q}_{l_i}{l}_i^b{x}_{l_i}^{-1}+{q}_{r_i}{r}_i^b{x}_{r_i}^{-1}\right)-{q}_{r_i}-{q}_{l_i}\right]}{A^2}},\end{array} $$

and

$$ \begin{array}{l}{L}_{12}=-{\displaystyle \sum_{i=1}^{n_1}\frac{t_i^b{\left( \log {t}_i\right)}^2}{{\left(1+{t}_i^b\right)}^2}}+2{\displaystyle \sum_{i={n}_1+1}^{n_1+{n}_2}\frac{F^2\left[{x}_l^{-a}{s}_{l_i}-{x}_r^{-a}{s}_{r_i}\right]}{A^3}}-{\displaystyle \sum_{i={n}_1+1}^{n_1+{n}_2}\frac{\left(a{q}_{r_i}{J}_{r_i}-a{q}_{l_i}^{-1}{J}_{l_i}\right)\left(-{x}_{r_i}^{-a}{s}_{r_i}+{x}_{l_i}^{-a}{s}_{l_i}\right)}{A^2}}\\ {}\kern1em +{\displaystyle \sum_{i={n}_1+1}^{n_1+{n}_2}\frac{q_{r_i}^2{r}_i^b{x}_{r_i}^{-a-2}\left[\left(a+1\right){r}_i^b-{x}_{r_i}^{-1}+a\left(a+1\right)\;{r}_i^b{s}_{r_i}{x}_{r_i}^{-a-2}+a\left(a+1\right){s}_{r_i}\right]}{A}}\\ {}\kern1em +{\displaystyle \sum_{i={n}_1+1}^{n_1+{n}_2}\frac{q_{l_i}^2{l}_i^b{x}_{l_i}^{-a-2}\left[\left(a+1\right){l}_i^b-{x}_{l_i}^{-1}+a\left(a+1\right)\;{l}_i^b{s}_{l_i}{x}_{l_i}^{-a-2}+a\left(a+1\right){s}_{l_i}\right]}{A}}\\ {}\kern1em +2a{\displaystyle \sum_{i={n}_1+1}^{n_1+{n}_2}\frac{\left[{q}_{r_i}{r}_i^b{x}_{r_i}^{-a-1}-{q}_{l_i}{l}_i^b{x}_{l_i}^{-a-1}\right]\left\{{q}_{r_i}{r}_i^b{x}_{r_i}^{-a-1}\left(a{s}_{r_i}-1\right)-{q}_{l_i}{l}_i^b{x}_{l_i}^{-a-1}\left(a{s}_{l_i}-1\right)\right\}}{A^2}},\end{array} $$

where

$$ B={s}_{r_i}-{s}_{l_i} $$, $$ {J}_r={r}_i^b{q}_{r_i}^2{x}_{r_i}^{-a-1}\left[\left(a+1\right){r}_i^b{x}_{r_i}^{-1}-1\right] $$ and $$ {J}_l={l}_i^b{q}_{l_i}^2{x}_{l_i}^{-a-1}\left[\left(a+1\right){l}_i^b{x}_{l_i}^{-1}-1\right] $$.

The elements *ε*_*ij*_ are obtained as follows:

$$ {\varepsilon}_{11}=-\frac{I}{D},{\varepsilon}_{12}={\varepsilon}_{21}=\frac{H}{D},{\varepsilon}_{22}=-\frac{G}{D} $$

where

$$ D=GI-{H}^2 $$

$$ \begin{array}{l}I=\frac{\partial^2L}{\partial {b}^2}=-\frac{\left({n}_1+\theta \right)}{b^2}-\left(a+1\right){\displaystyle \sum_{i=1}^{n_1}\frac{t_i^b{\left( \log {t}_i\right)}^2}{{\left(1+{t}_i^b\right)}^2}}-{\displaystyle \sum_{i={n}_1+1}^{n_1+{n}_2}\frac{F^2}{A^2}}\\ {}+{\displaystyle \sum_{i={n}_1+1}^{n_1+{n}_2}\frac{a{q}_{r_i}^2\left[\left(a+1\right){x}_{r_i}^{-a-2}{r}_i^{2b}-{r}_i^b{x}_{r_i}^{-a-1}\right]+a{q}_{l_i}^2\left[-\left(a+1\right){x}_{l_i}^{-a-2}{l}_i^{2b}+{l}_i^b{x}_{l_i}^{-a-1}\right]}{A}},\end{array} $$

$$ \begin{array}{l}H=\frac{\partial^2L}{\partial a\partial b}=-1-{\displaystyle \sum_{i=1}^{n_1}\frac{t_i^b\left( \log {t}_i\right)}{\left(1+{t}_i^b\right)}}+{\displaystyle \sum_{i={n}_1+1}^{n_1+{n}_2}\frac{x_{r_i}^{-a-1}{r}_i^b{q}_{r_i}\left(a{s}_{r_i}-1\right)-{x}_{l_i}^{-a-1}{l}_i^b{q}_{l_i}\left(a{s}_{l_i}-1\right)}{A}}\\ {}-{\displaystyle \sum_{i={n}_1+1}^{n_1+{n}_2}\frac{\left(-{x}_{r_i}^{-a}{s}_{r_i}+{x}_{l_i}^{-a}{s}_{l_i}\right)\left(-a{q}_{r_i}{r}_i^b{x}_{r_i}^{-a-1}+a{q}_{l_i}{l}_i^b{x}_{l_i}^{-a-1}\right)}{A^2}},\end{array} $$

and

$$ G=\frac{\partial^2L}{\partial {a}^2}=-\frac{\left({n}_1+\theta -1\right)}{a^2}-{\displaystyle \sum_{i={n}_1+1}^{n_1+{n}_2}\frac{x_{l_i}^{-a}{x}_{r_i}^{-a}{B}^2}{A^2}}. $$

where

$$ F=a{q}_{l_i}{l}_i^b{x}_{l_i}^{-a-1}-a{q}_{r_i}{r}_i^b{x}_{r_i}^{-a-1} $$

The Bayes estimator of the function *U* (*a*, *b*) under the SEL function, given by Lindley’s method in (3.5), turn out to be:

$$ \widehat{U}{}_s=U\left(a,b\right)-\frac{W}{2D}+\frac{\varPsi_1}{2{D}^2}{u}_1+\frac{\varPsi_2}{2{D}^2}{u}_2 $$

where

$$ \begin{array}{l}W={u}_{11}I-H\left({u}_{12}+{u}_{21}\right)+{u}_{22}G,\\ {}{\varPsi}_1={L}_{30}{I}^2+{L}_{12}\left(GI+2{H}^2\right)-3{L}_{21}HI-{L}_{03}GH,\\ {}{\varPsi}_2=-{L}_{30}IH+{L}_{21}\left(GI+2{H}^2\right)-3{L}_{12}GH+{L}_{03}{G}^2.\end{array} $$

If *U*(*a*, *b*) = *a* then

4.7 $$ \widehat{a}{}_S=\widehat{a}{}_M+\frac{\varPsi_1}{2{D}^2}. $$

If *U*(*a*, *b*) = *b* then

4.8 $$ \widehat{b}{}_S=\widehat{b}{}_M+\frac{\varPsi_2}{2{D}^2}. $$

## Simulation results

This section presents the numerical results for evaluating the performance of the two estimation methods for different sample size and censoring schemes. The author wrote R- subroutine to conduct the simulation study and it is available upon request.

Five different sample sizes viz *n* =10, 30, 50, 70 and 100 with five combination of the censoring schemes (*λ*^− 1^, *γ*^− 1^) =(0.25,0.25), (0.5,0.5), (0.5,0.75), (1,0.75) and (1.25,0.5). For all considered cases and without loss of generality, the random samples with desired sizes are generated from the Burr-XII distribution with parameters *a* = 1 and *b* = 1 are middle-censored according to (1.1). The MLE based on the iterative procedure given in (3.2, 3.3) and the Bayes estimates with respect to SEL and using the prior gamma with *θ* = 0.1 and *β* = 0.1 are obtained using Equations (4.6, 4.7 and 4.8).

For each combination of sample size and censoring scheme the process is repeated 1000 times and the average estimates, the mean squared error (MSE) within brackets and the average censoring percentage (*CP*) are obtained and reported in Table 1.

**Table 1:** **Average estimates and the corresponding MSE (within brackets) of two estimators**

***n***	**Method**	**(0.25,0.25)**		**(0.5,0.5)**		**(0.5,0.75)**		**(1,0.75)**		**(1.25,0.5)**	
		***a***	***b***	***a***	***b***	***a***	***b***	***a***	***b***	***a***	***b***
10	MLE	1.156	1.095	1.144	1.147	1.136	1.081	1.104	1.136	1.088	1.112
		(0.171)	(0.107)	(0.159)	(0.162)	(0.150)	(0.092)	(0.116)	(0.150)	(0.099)	(0.125)
	Bayes	1.104	1.079	1.097	1.113	1.133	1.074	1.087	1.130	1.084	1.096
		(0.130)	(0.102)	(0.122)	(0.141)	(0.163)	(0.096)	(0.111)	(0.159)	(0.108)	(0.121)
	*CP*	19.5%		25.2%		33.4%		24.3%		15.8%	
30	MLE	1.098	1.079	1.073	1.082	1.102	1.071	1.074	1.088	1.080	1.067
		(0.031)	(0.032)	(0.030)	(0.033)	(0.034)	(0.039)	(0.030)	(0.036)	(0.033)	(0.037)
	Bayes	1.039	1.036	1.064	1.072	1.100	1.049	1.072	1.080	1.077	1.037
		(0.034)	(0.036)	(0.032)	(0.038)	(0.039)	(0.040)	(0.036)	(0.037)	(0.034)	(0.030)
	*CP*	15.1%		20.2%		29.3%		20.1%		13.3%	
50	MLE	1.018	1.017	1.008	1.032	1.061	1.036	1.052	1.062	1.022	1.022
		(0.028)	(0.026)	(0.029)	(0.025)	(0.029)	(0.026)	(0.025)	(0.022)	(0.025)	(0.027)
	Bayes	1.036	1.032	1.049	1.051	1.094	1.036	1.066	1.071	1.049	1.035
		(0.030)	(0.031)	(0.029)	(0.033)	(0.031)	(0.032)	(0.028)	(0.032)	(0.028)	(0.022)
	*CP*	14. 9%		19.7%		29.5%		21.4%		14.1%	
70	MLE	0.990	0.962	0.997	1.010	1.031	0.997	0.982	1.037	0.985	0.996
		(0.013)	(0.012)	(0.015)	(0.013)	(0.017)	(0.017)	(0.010)	(0.018)	(0.016)	(0.017)
	Bayes	1.015	0.974	1.008	1.025	1.048	1.034	1.042	1.053	1.002	1.012
		(0.016)	(0.015)	(0.019)	(0.017)	(0.021)	(0.022)	(0.015)	(0.021)	(0.020)	(0.021)
	*CP*	15.2%		20.3%		28.8%		20.9%		13.9%	
100	MLE	0.961	0.958	0.993	0.996	0.978	0.983	0.929	1.002	0.907	0.971
		(0.010)	(0.010)	(0.009)	(0.011)	(0.009)	(0.012)	(0.008)	(0.012)	(0.013)	(0.013)
	Bayes	1.001	0.962	1.012	1.015	0.998	0.995	0.980	1.020	0.980	0.981
		(0.012)	(0.013)	(0.011)	(0.013)	(0.015)	(0.014)	(0.015)	(0.016)	(0.017)	(0.016)
	*CP*	15.0%		20.5%		28.9%		21.1%		14.5%	

*CP* is the mean of censoring percentages.

Results in Table 1 show that both MLE and Bayes estimates behave almost similarly. For all censoring schemes, there is a decreasing function between the sample size and both of the average bias and the mean squared error, which verifies the consistency property of the both estimates. The mean censoring percentages are highly affected by the censoring parameters, with insignificant effect on the average estimates.

For further investigation of the properties of the MLE based on the approximated Fisher information matrix (3.4), the average lengths of the 95% confidence interval is computed as well as the corresponding coverage percentage within brackets are given in Table 2.

**Table 2:** **The average lengths of the confidence interval and the corresponding coverage percentages (with brackets)**

***n***	**(0.25,0.25)**		**(0.5,0.5)**		**(0.5,0.75)**		**(1,0.75)**		**(1.25,0.5)**	
	***a***	***b***	***a***	***b***	***a***	***B***	***a***	***b***	***a***	***b***
10	1.376	1.353	1.503	1.368	1.538	1.346	1.351	1.412	1.404	1.289
	(0.96)	(0.97)	(0.97)	(0.96)	(0.97)	(0.97)	(0.96)	(0.96)	(0.97)	(0.96)
30	0.703	0.737	0.748	0.796	0.767	0.725	0.730	0.796	0.777	0.784
	(0.96)	(0.96)	(0.96)	(0.96)	(0.96)	(0.96)	(0.96)	(0.96)	(0.96)	(0.96)
50	0.583	0.515	0.567	0.533	0.539	0.582	0.582	0.510	0.538	0.495
	(0.94)	(0.95)	(0.94)	(0.95)	(0.95)	(0.96)	(0.95)	(0.95)	(0.94)	(0.95)
70	0.416	0.441	0.479	0.470	0.468	0.452	0.448	0.442	0.463	0.476
	(0.96)	(0.95)	(0.96)	(0.96)	(0.95)	(0.95)	(0.95)	(0.94)	(0.95)	(0.95)
100	0.356	0.349	0.368	0.371	0.358	0.345	0.375	0.317	0.328	0.368
	(0.95)	(0.95)	(0.94)	(0.95)	(0.95)	(0.95)	(0.94)	(0.95)	(0.95)	(0.94)

Results in Table 2 show that the coverage percentages are very close to the nominal level (95%), with slight variation for small sample size (*n* =10). There is an inverse relationship between the average lengths of the confidence interval and sample size.

## Data analysis

For the illustrative purpose, we consider a real data set which was generated from a clinical trial describing a relief time (in hours) for 50 arthritic patients as given in Wingo (1993) who showed that the Burr-XII model can not be rejected to fit the data. The data were also analyzed by different authors Wu et al. (2010) and Soliman et al. (2011).

The arthritic data were artificially middle-censored by considering that the left end was an exponential random variable with mean 0.3 and the width was exponential with mean 0.3. Then the data were rearranged and given below:

Data set: 0.29, 0.29, 0.34, 0.34, 0.35, 0.36, 0.36, 0.36, 0.44, 0.44,0.46, 0.46, 0.49, 0.49, 0.50, 0.50, 0.54, 0.55, 0.55, 0.55, 0.56, 0.57, 0.58, 0.59, 0.59, 0.60, 0.60, 0.61, 0.61, 0.62, 0.68, 0.70, 0.70, 0.71, 0.71, 0.71, 0.72, 0.73, 0.75, 0.75, 0.80, 0.81, 0.82, 0.84, 0.84, 0.87, (0.36, 0.80), (0.53,1.14), (0.50, 0.74), (0.60, 0.91).

There are four middle-censored observations are listed at the end of the data set, where *n*_1_ = 46 and *n*_2_ = 4 with censoring percentage 8.69%. The MLE of scale and shape parameters are *â* =7.423 and $\widehat{b}$ =4.654 with 95% confidence interval based on the asymptotic distributions *â* and $\widehat{b}$ are (7.402, 7.443) and (4.395, 4.913) respectively. The Bayes estimates of *a* and *b* are 7.628 and 4.157, respectively.

## Conclusions

The analysis of Burr XII distribution with middle-censoring was considered, where the parameter estimates were obtained by the maximum likelihood based on iterative procedures and Bayesian methods using the Lindley’s approximation. Both estimators behave almost similarly and verified the consistency property. Several related open problems would be interesting to be considered such as exploring the middle-censoring of Burr-XII model of covariates.
